# ANLN promotes carcinogenesis in oral cancer by regulating the PI3K/mTOR signaling pathway

**DOI:** 10.1186/s13005-021-00269-z

**Published:** 2021-06-03

**Authors:** Bing Wang, Xiao-li Zhang, Chen-xi Li, Ning-ning Liu, Min Hu, Zhong-cheng Gong

**Affiliations:** 1 Oncological Department of Oral and Maxillofacial Surgery, Xinjiang Medical University Affiliated First Hospital, Stomatological School of Xinjiang Medical University, Stomatology Research Institute of Xinjiang Province, No.137 Liyushan South Road, 830054 Urumqi, PR China; 2 People’s Hospital of Xinjiang Uygur Autonomous Region, 830001 Urumqi, PR China; 3 Department of Oral and Maxillofacial Surgery, Laboratory for Tumor Genetics and Regenerative Medicine, The Head and Neurocenter, University Hospital Hamburg-Eppendorf (UKE), Martinistrasse 52, 20246 Hamburg, Germany; 4 Department of Prosthodontia, Xinjiang Medical University Affiliated First Hospital, 830054 Urumqi, PR China; 5 Urumqi Myour Dental Clinic, 830002 Urumqi, PR China

**Keywords:** ANLN, Oral cancer, mTOR, PI3K signaling pathway

## Abstract

**Background:**

Oral cancer is a malignant disease that threatenshuman life and greatly reducespatientquality of life. ANLN was reported to promote the progression of cancer. This study aims to investigate the role of ANLNin oral cancer and the underlying molecular mechanism.

**Methods:**

ANLN expression was downregulated by RNAi technology. The effect of ANLN on cell behaviors, including proliferation, cell cycle progression, invasion, and apoptosis, was detected. Western blotting analysis was used to explore the mechanism by whichANLN functions in oral cancer.

**Results:**

Data from TCGA database showed that ANLN was expressed at significantly higher levels in tumor tissues thanin normal control tissues. Patients with higher ANLN expression exhibitedshorter survivaltimes. ANLN was alsoabundantly expressedin the cancer cell lines CAL27 and HN30. When ANLN was knocked down in CAL27 and HN30 cells, cell proliferation and colony formation weredecreased. The cell invasion ability was also inhibited. However, the cell apoptosis rate was increased. In addition, the levels of critical members of the PI3K signaling pathway, includingPI3K, mTOR, Akt, and PDK-1, were significantlyreducedafter ANLN was knocked down in CAL27 cells.

**Conclusions:**

ANLN contributes to oral cancerprogressionand affects activation ofthe PI3K/mTOR signaling pathway. This study providesa new potential targetfor drug development and treatment in oral cancer.

## Background

Actin is an important moleculethat is responsible for cell motility and muscle cellcontraction. Anillin actin-binding protein (ANLN) is an actin-binding protein thatplays critical roles in cell growth, migration, and cytoskeletal dynamics in podocytes [1]. The ANLN gene has beenmapped to location 7p14.2 in the human genome. Mutations in ANLN have beendetected in patients with focal segmental glomerulosclerosis, and additionalexperiments have suggested that ANLN contributes to tumor progression [2]. For example, overexpression of ANLN promoted cell metastasis in lung adenocarcinoma, and patients with higher ANLN expression displayed much worse prognosis [3, 4]. Overexpression of ANLN was observedin pancreatic cancer,anddownregulation of ANLN by microRNA inhibited cell aggressiveness [5, 6]. In cervical cancer, bioinformatics analysis demonstrated that ANLN might be a candidate biomarker for the evaluation of survival [7]. Moreover, ANLN increased theresistance of breast cancer cells to chemotherapy and reduced the effectiveness of doxorubicin or anthracycline in the clinic [8, 9]. Based on large published profiles, ANLN promotesthe progression ofcancer and might be a novel target forcancer therapy [10].

Oral cancer is a subtype of head and neck squamous cell carcinoma that accounts for approximately 2 % of all cancer cases, and the mortalityrate is approximately50 % [11]. Patients with localized disease benefit from currenttherapies, including surgery, chemotherapy and radiotherapy. However,the prognosisof patients with metastatic disease is poor. Thetumor stage of patients is a factor that substantially affects the survival rate. The overall 5-year survival rate is approximately 80 % for stage I patients but 40 % for stage IV patients [11, 12]. Moreover, the quality of life of patients is greatly decreased due toabnormal speech, cosmetic appearance and eating habits [13]. Therefore, it is urgent to elucidatethe pathogenesis of oral cancer and the underlying molecular mechanism.

ANLN was reported to promote the progression ofmany cancers, but its function in oral cancer is not known. Shimizu reported that the mRNA level of ANLN was upregulated in head and neck squamous cell carcinoma [14]. However, no further information has beenpublished. In this study, to investigate the role of ANLN in oral cancer, we reduced the expression of ANLN in oral cancer cells and assessedproliferation, aggressiveness, apoptosis, and cell cycle progression in oral cancer cells. In addition, the signaling pathway regulated by ANLN was preliminarily explored.

## Methods

### Cell lines and cell culture

The human oral cancer cell lines HN30 and CAL27 were purchased from the Chinese Academy of Sciences (Shanghai, China) and cultured in DMEM (Gibco, CA, USA) containing 10 % FBS (Gibco, CA, USA) and 1 % penicillin-streptomycin (Beyotime, Shanghai, China).

### Synthesis of small interfering RNA oligonucleotides and transfection

Two small RNA oligonucleotide fragments (siRNA-#1 and siRNA-#2) targeting the human ANLN gene were designed and synthesized. The target sequence for the human ANLN gene is listed in Table 1. Then, Lipo3000 reagent was used to transfer the two siRNAs into oral cancer cells. In brief, 1 µL of Lipo3000 (Invitrogen, CA, USA) was mixed with 50 pmol siRNA and incubated for 20 min at room temperature. Then, the mix was added to prepared cells in 24-well plates. After 6 h of culture, the supernatant was removed, and new fresh medium was added. Then, the cells were cultured for another 48 h.

**Table 1:** Target sequence for knockdown of the human ANLN gene

Gene symbol	Name	Target sequence (5′-3′)
ANLN	SiRNA-1	GGACAAAGACACAAGCAAA
	SiRNA-2	GGAAAAAGAAGAAAAGACA

### Real-time quantitative polymerase chain reaction assay (qPCR)

Total RNA was extracted from cultured cells with an RNAeasy Kit (Beyotime, Shanghai, China) according to the manufacturer’s instructions. Briefly, the cells were lysed with lysis buffer, and binding buffer was added to the lysis mixture. Then, the mixture was transferred to a collection column and washed with wash buffer A/B. Finally, RNA was eluted with elution buffer and quantified withan ultraviolet spectrophotometer (Onedrop1000, Hangzhou, China).

Approximately0.5 µg of total RNA was used to synthesize first-strand cDNA with a cDNA synthesis kit (Yeasen, Shanghai, China). Then,1 µL cDNA was used for the qPCR assay with SYBR Green mix (Beyotime, Shanghai, China) on an ABI 7000 system (Applied Biosystems, USA). The cycling conditionsfor the qPCR assay wereas follows: 95 °C, 5 min; (95 °C, 10 s and60°C, 15 s) for 40 cycles. β-actin was chosen as the internal control. The relative mRNA expression level of each gene was calculated by the 2^−△△CT^ method. The primers are shown in Table 2.

**Table 2:** Primers for the quantificationof human ANLN gene expression

Gene symbol	Primer	Sequence (5′-3′)	Product
ANLN	Forward	AGTCCTCTGAAAACGGGGGT	214 bp
	Reverse	GTGTGCTACGAGCTGGACTT	

### Western blotting assay

Total protein was extracted from cultured cells with a protein extraction kit (Beyotime, Shanghai, China) according to the manufacturer’s instructions. In brief, the cells were collected by centrifugation at 1200 rpm for 5 min at room temperature. The cell pellet wasresuspended in lysis buffer, transferred to a protein column and washed with serial buffer. Finally, the proteins were eluted with elution buffer, and the total protein concentration was detected withan ultraviolet spectrophotometer (Onedrop1000, Hangzhou, China).

Ten micrograms of total protein was separated on 15 % SDS-PAGE gels for 1.5 h. Then,the proteins were transferred toPVDF membranes (Beyotime, Shanghai, China) and analyzed by western blotting analysis. In brief, the PVDF membranes were blocked with 1 % BSA for 1 h at room temperature and washed with PBS three times. Then, a primary antibody against each targetprotein was added to the PVDF membranes and incubated for 12 h at 4 °C. After washing with PBS three times, the PVDF membranes were incubated with the secondary antibody for 2 h at room temperature. Finally, after the PVDF membranes were washed with PBS, each targetprotein was detected by an ECL kit (Yeasen, Shanghai, China) according to the manufacturer’s instructions. The following primary antibodies were used: PI3K (ab140307, 1:2000, Abcam, CA, USA), mTOR (ab134903, 1:6000, Abcam, CA, USA), AKT (ab18785, 1:1000, Abcam, CA, USA), PDK-1 (ab207450, 1:2000, Abcam, CA, USA), and GAPDH (ab9485, 1:2000, Abcam, CA, USA).

### Cell proliferation assay

Oral cancer cells treated with siRNA were seeded into a 96-well plate at a density of 5 × 10^3^cells/100µL/well and cultured for 96 h. Then,CCK-8 reagent was added to each well (10 % of the total volume in the well) every 24 h, and the cells were cultured for another 1 h. The absorbance value at a wavelength of 450 nm was detected on a microplate reader (Tecan, Switzerland). Each experiment was repeated three independenttimes.

### Plate colony formation

Oral cancer cells treated with siRNA were seeded into a 24-well plate at a density of 0.5 × 10^3^ cells/100µL/well and cultured for 14 days. After 14 days, the cells were fixed in 4 % paraformaldehyde (Beyotime, Shanghai, China) for 15 min and washed with PBS three times. Then,the cells were stained in 0.5 % crystal staining buffer (Sangon, Shanghai, China) for 15 min at room temperature. After washing with PBS three times, positively stained cells were countedunder a microscope (Olympus, Tokyo, Japan). Each experiment was repeated three independenttimes.

### Transwell assay

Transwell inserts with 8-µm pores were treated with 200 µL Matrigel (BD Science, USA) and plated overnight. After washing with 200 µL culture medium, oral cancer cells treated with siRNA were seeded into the insert at a density of 1 × 10^4^ cells/300µL/well and placed into a 24-well plate. Culture medium with no FBS was added to the upper chamberof the insert andculture medium with 10 % FBS was added to the lower chamber. After culturing for 48 h, the cells in the upper chamberof the insert were gentlyscraped, and the cells in the lower chamber were fixed in 4 % paraformaldehyde (Beyotime, Shanghai, China) for 15 min and washed with PBS three times. Then, the cells were stained in 0.5 % crystal staining buffer (Sangon, Shanghai, China) for 15 min at room temperature. After washing with PBS three times, the positively stained cells were countedunder a microscope (Olympus, Tokyo, Japan). Each experiment was repeated three independenttimes.

### Cell apoptosis detection

Oral cancer cells treated with siRNA were seeded into a 6-well plate at a density of 2 × 10^5^ cells/500µL/well and cultured for 48 h. Then,the cells were treated with an apoptosis detection kit (Beyotime, Shanghai, China). In brief, the cells were collected by centrifugation at 1200 rpm for 5 min at room temperature. Then,the cells were resuspended in 100 µL staining buffer containing 5 µL Annexin V/FITC and 10 µL PI reagent. After incubationfor 15 min at room temperature in the dark, 400 µL staining buffer was added, and cell apoptosis was analyzed on a flow cytometer (BD, FACSCelesta, CA, USA). Each experiment was repeatedthree independenttimes.

### Cell cycle detection

Oral cancer cells treated with siRNA were seeded into a 6-well plate at a density of 2 × 10^5^ cells/500µL/well and culture for 48 h. Then,the cells were treated with a cell cycle detection kit (Yeasen, Shanghai, China). In brief, the cells were collected by centrifugation at 1200 rpm for 5 min at room temperature. Then,the cells were resuspended in 500 µL staining buffer containing 10 µL RNase A and 10 µL PI reagent. After incubationfor 30 min at room temperature in the dark, cell cycle progressionwas analyzed on a flow cytometer (BD, FACSCelesta, CA, USA). Each experiment was repeated three independenttimes.

### Statistical analysis

All the statistical analyses were carried out with SPSS 16.0 software. All the data are displayed as the mean value plus the standard variation (mean ± sd). In brief, the difference between two groups was analyzed by unpaired Student’s t test. One-way ANOVA was used to analyze the differences among multiple groups. A *P* value less than 0.05 was considered to indicate statistically significant differences.

## Results

### ANLN expression is associated with survival in patients with oral cancer

**Fig. 1:**
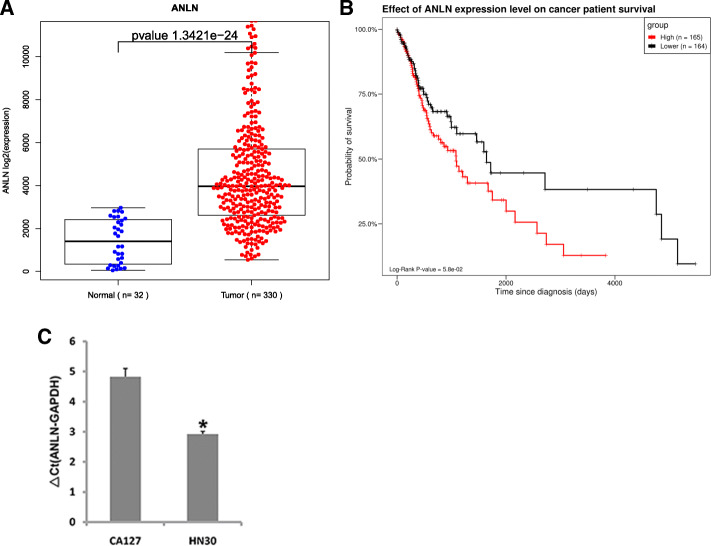
The expression pattern of ANLN in oral cancer. **a** ANLN was overexpressed in tumor tissues compared to adjacent normal tissues. **b** The survival rate of patients with high ANLN expression was worse than that of patients with low ANLN expression. **c** ANLN was highly expressed in oral cancer cell lines. *P<0.05 indicates significance. Each experiment was repeated at least three times

To evaluate the clinical significance of ANLN in oral cancer, ANLN expression data and clinical profiles were downloaded from TCGA database. As shown in Fig. 1 a, ANLN expression was significantly higher in tumor tissues than in adjacent normal control tissues. The 5-year survival rate of patients with high ANLN expression was much worse than that of patients with low ANLN expression (*p*<0.05) (Fig. 1b). In addition, ANLN was abundantly expressed in the tumor cell lines CAL27 and HN30 (Fig. 1 c). These data suggest that ANLN is important in oral cancer.

### ANLN contributes to cell growth and proliferation

**Fig. 2:**
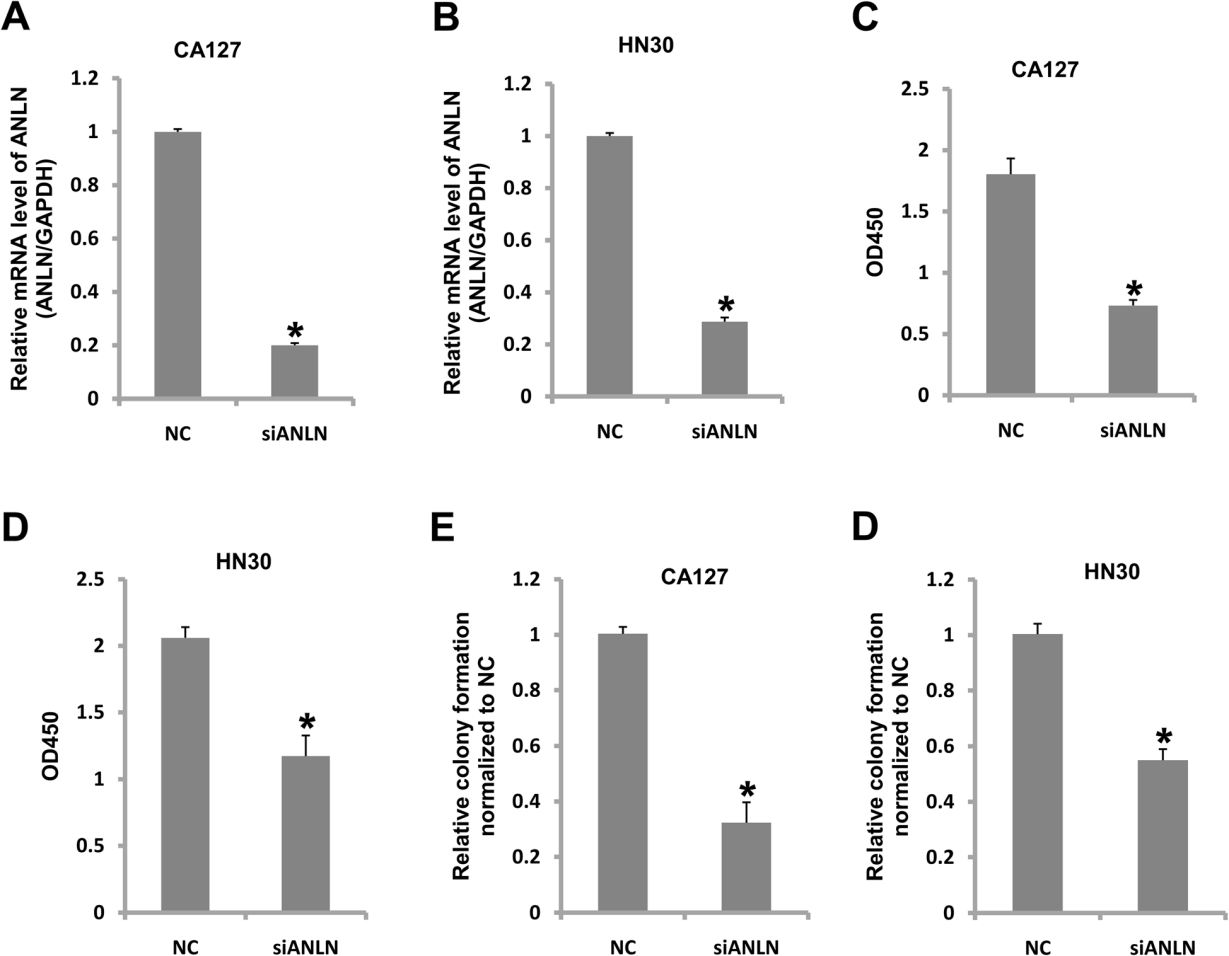
Knockdown of ANLN inhibited cell growth and proliferation in oral cancer. **a** ANLN was efficiently knocked down in CAL27 cells. **b** ANLN was efficiently knocked down in HN30 cells. **c** Knockdown of ANLN inhibited CAL27 cell proliferation. **d** Knockdown of ANLN inhibited HN30 cell proliferation. **e** Knockdown of ANLN inhibited CAL27 cell colony formation. **f** Knockdown of ANLN inhibited HN30 cell colony formation. *P<0.05 indicates significance. Each experiment was repeated at least three times

To investigate the role of ANLN in oral cancer, the expression of ANLN in CAL27 and HN30 cells was reduced by RNAi technology. The mRNA level of ANLN was reduced by 80 % in CAL27 cells and 72.3 % in HN30 cells (Fig. 2 a and b). The cell proliferation rate in CAL27 cells was decreased by 59.3 %, and it was decreased by 44 % in HN30 cells (Fig. 2 c and d). The colony formation ability was reduced by 67.7 % in CAL27 cells,andit was reduced by 45 % in HN30 cells (Fig. 2e, f). Therefore, ANLN contributes to cell growth and proliferation in oral cancer.

### ANLN is not necessary forcell cycle progression

**Fig. 3:**
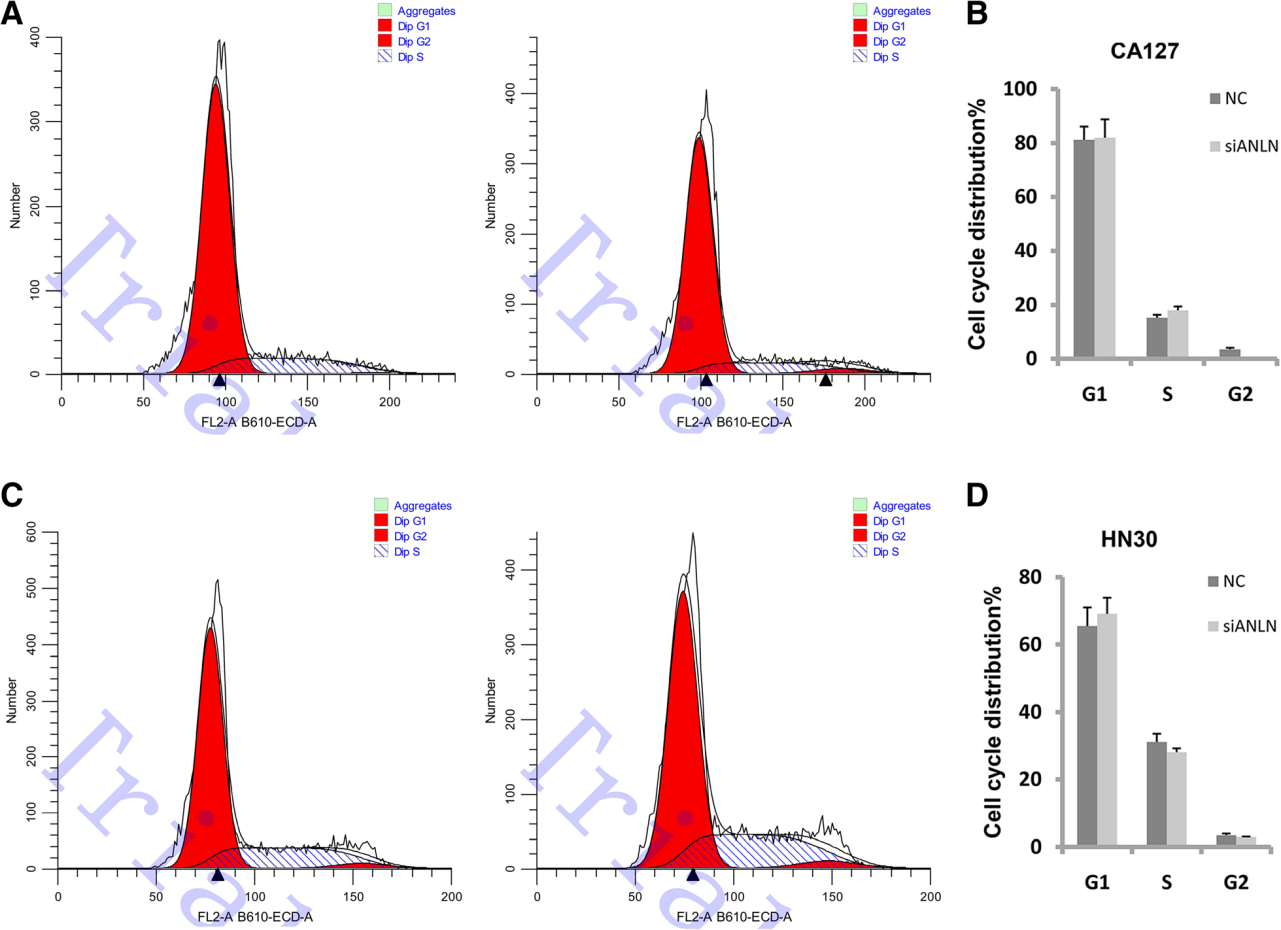
ANLN affected cell cycle transition in oral cancer. **a** Cell cycle progression of CAL27 cells after ANLN expression was reduced. **b** Analysis of data in A. **c** Cell cycle progression of HN30 cells after ANLN expression was reduced. **d** Analysis of data in C. Each experiment was repeated at least three times

PI staining was used to detect cell cycle progressionin cells in whichANLN was knocked down. As shown in Fig. 3 a and b, the number of CAL27 cells in the G1 phase slightly increased when ANLN expression was reduced. In HN30 cells, the number of cellsin the G1 phase also slightly increased (Fig. 3 c and d). In both cell lines, ANLN knockdown blocked cell cycle progressionfrom the G1 phase to the S phase, but the effect was not significant, which suggests that ANLN has little influence on cell cycle progression in oral cancer.

### Cell invasion is inhibitedby ANLN knockdown

**Fig. 4:**
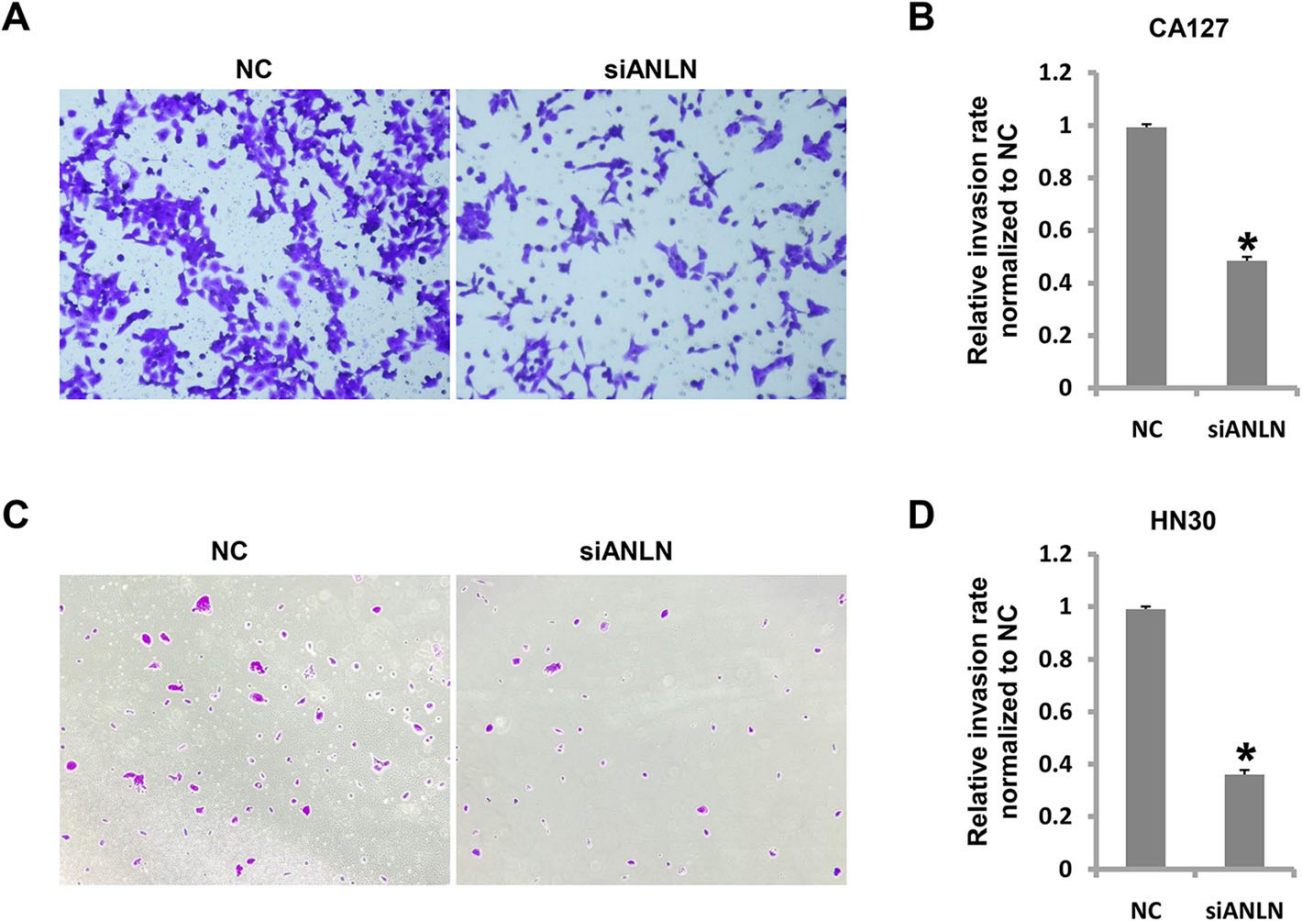
ANLN was essential to oral cancer cell invasion. **a**.CAL27 cell invasion was suppressed after ANLN expression was reduced. **b** Analysis of data in **a**. **c** HN30cell invasion was suppressed after ANLN expression was reduced. **d** Analysis of data in **c**. *P<0.05 indicates significance. Each experiment was repeated at least three times

To detect the effect of ANLN knockdown on the invasion of oral cancer cells, a Transwell assay was conducted. As shown in Fig. 4 a and b, the relative cell invasion rateof CAL27 cells was 48.7 % when ANLN expression was reduced. Consistently, the relative cell invasion rate was 36.4 % in HN30 cells (Fig. 4 c and d). In both cell lines, the invasive ability was suppressed when ANLN expression was reduced. These findingssuggest that ANLN might be a critical factor in the metastasis oforal cancer.

### Cell apoptosis is induced by ANLN knockdown

**Fig. 5:**
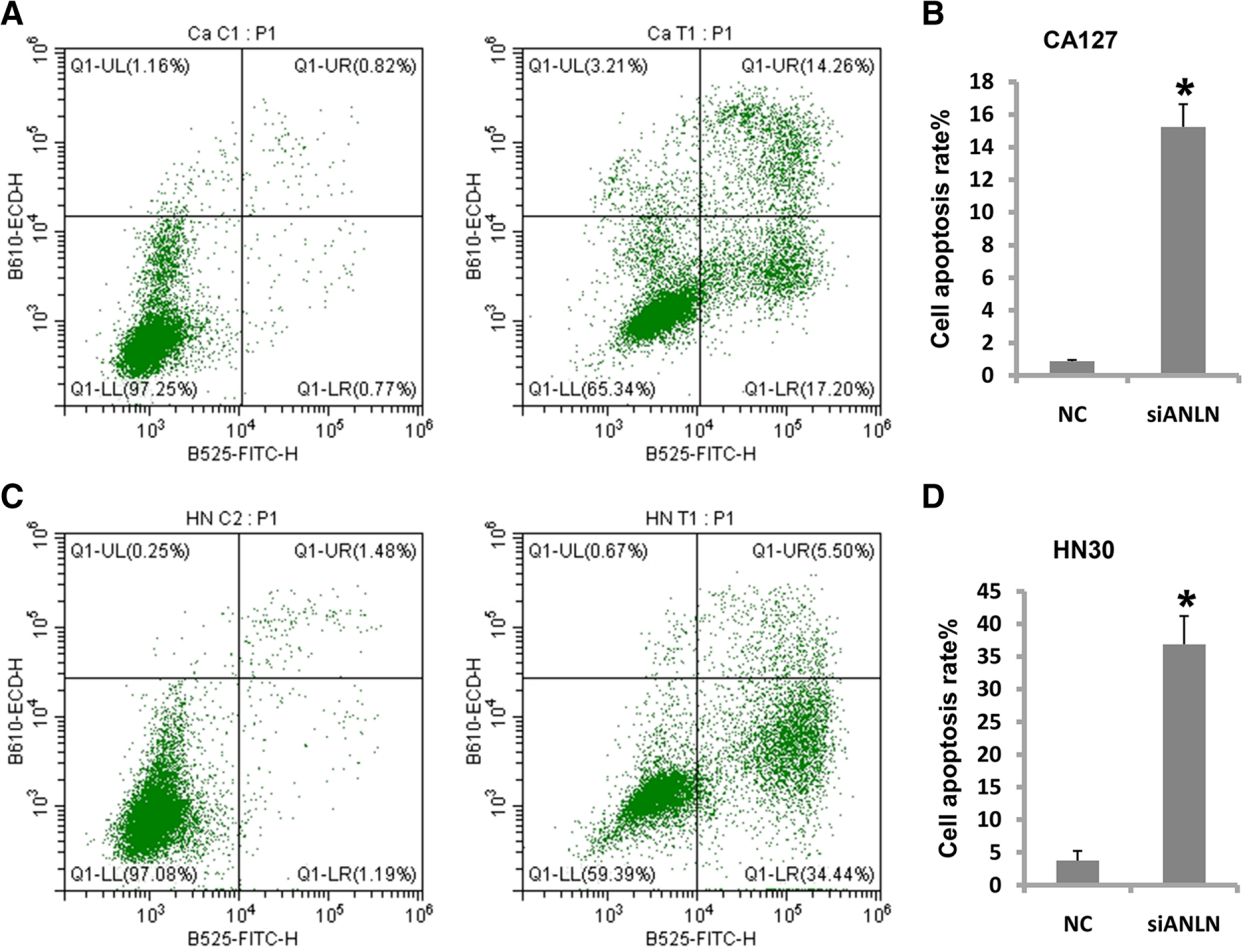
Knockdown of ANLN induced cell apoptosis in oral cancer. **a** Apoptosis of CAL27 cells was induced after ANLN expression was reduced. **b** Analysis of data in **a**. **c** HN30 cell apoptosis was induced after ANLN expression was reduced. **d** Analysis of data in **c**. *P<0.05 indicates significance. Each experiment was repeated at least three times

Resistance to programmed cell death or apoptosis is common in tumors. Double staining with FITC and PI dye was usedto detect apoptosis in oral cancercells. The cell apoptosis rate increased from 0.88 to 15.24 % when ANLN expression was reduced in CAL27 cells (Fig. 5 a and b). Consistently, the cell apoptosis rateincreased from 3.72 to 36.88 % in HN30 cells (Fig. 5 c and d). In conclusion, reduced ANLN expression induced cell apoptosis in both CAL27 and HN30 cells, which suggests that ANLN inhibits cell apoptosis in oral cancer.

### PI3K/mTOR signaling is activated by ANLN

**Fig. 6:**
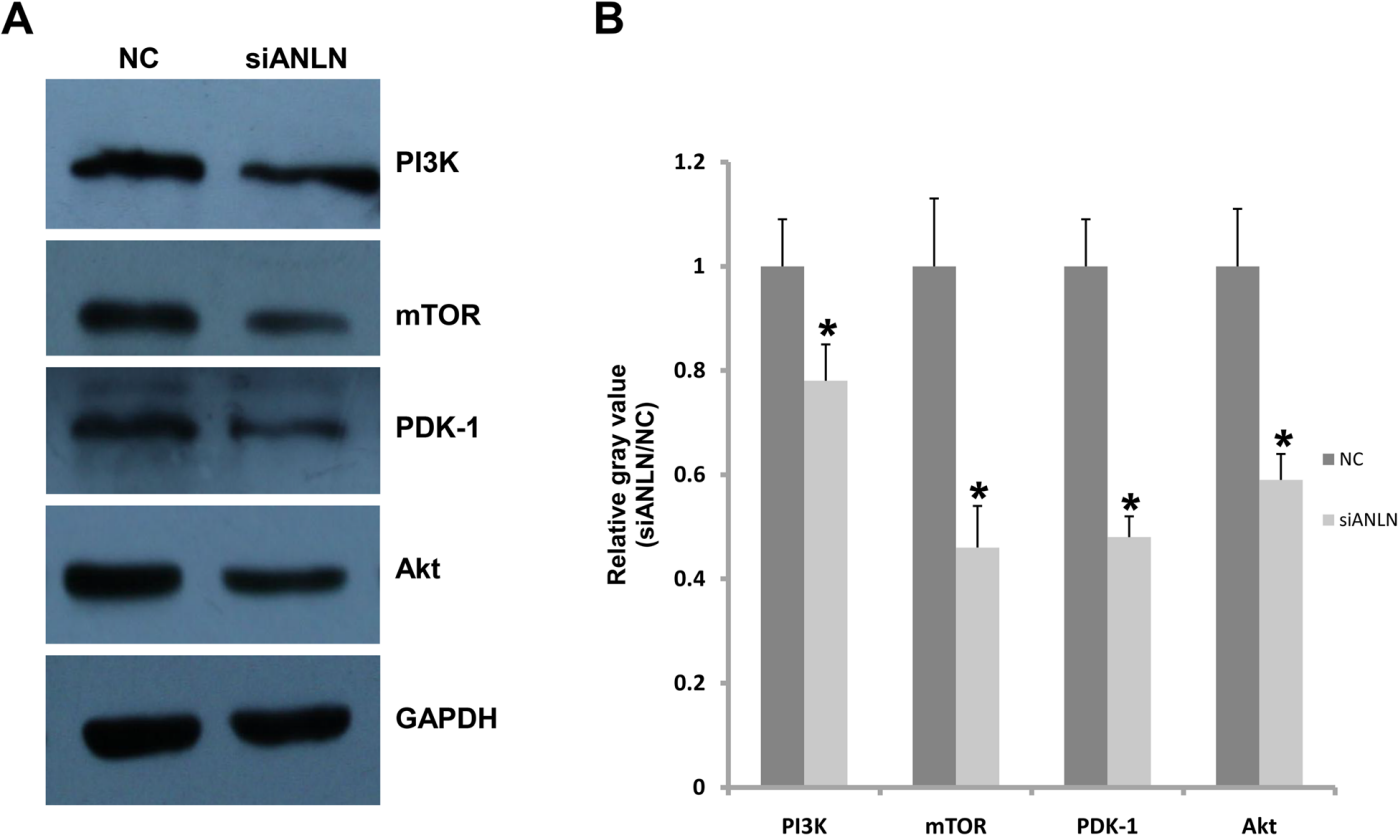
ANLN regulated the activation of the PI3K/mTOR signaling pathway. **a** Western blotting analysis showed that the expression of PI3K, mTOR, PDK-1, and Akt was decreased after ANLN expression was reduced in CAL27 cells. **b** Analysis of gray value analysis data in **a**. *P<0.05 indicates significance. Each experiment was repeated at least three times

PI3K/mTOR signaling plays an important role in cell survival and proliferation. In this study, the expression of PI3K, mTOR, and Akt was regulated by ANLN in CAL27 cells. As shown in Fig. 6 a, reduced ANLN expression in CAL27 cells decreased the expression levels of PI3K, mTOR, PDK1, and Akt. The protein level of PI3K decreased by 22 % after ANLN was knocked down. ANLN knockdown resulted in 54 %, 52 %, and 41 % decreases inmTOR, PDK1, and Akt expression, respectively (Fig. 6b). These molecules are key members of thePI3K/mTOR signaling pathway. Therefore, ANLN contributes to the activation of PI3K/mTOR signaling in oral cancer.

## Discussion

In recent years, major advances in the treatment of oral cancer have been made. Systemic therapies, such as chemotherapy and immunotherapy,yielded positive improvement for patients with oral cancer [11, 12, 15]. However, damage to normal tissues is a major problemofsystemic therapy and will be a substantial challenge for a very long time. In addition tononspecific adverse effects, the abnormalspeech, eating habits and social interactions caused by oral cancer greatly reduces patients’ quality of life [13]. According to a previous study, the overall survival rate of patients with oral cancer was dependent on tumor stage [11, 12]. Patients with higher stages displayed worse survival in the clinic. Therefore, early diagnosis is critical for the treatmentand prognosis oforal cancer patients. Unfortunately, oral cancer is a heterogeneous disease that leads to different responses to a particular treatment [16]. Accordingly, it is thoughtthat the identification of specific markersfor particular stages will be helpful for the treatment ofin oral cancer.

Based on the large amount of data from the PubMed database, a series of genes have been shown to play critical roles in oral cancer. Advances in biotechniques and medical knowledge are a major factor, but variance in tumor genetics also plays a certain role. Genome instability is a common phenomenon in cancer [17]. In each cancer, variation in several dozens of genescan be detected. In a particular cohort of patients, one or two gene variances might determineprogression or metastasis. ANLN is an actin-binding proteinresponsible for cell motility [1]. Multiple studies haveshownthat ANLNexpression is associated with poor survival in cancer patients [2]. In this study, we analyzed ANLNexpression data in TCGA database and found that ANLN was overexpressed in oral cancer specimens compared to adjacent normal control specimens. Higher ANLN expression was associated with shorter survival time. These data further suggested that ANLN expression is associated with poor prognosis in cancer patients and increase the list of cancers affected by ANLN.

Based on public data, ANLN can promote cell proliferation and growth in cancer and increase cell migration and invasion [2–7]. Our data were consistent with a previous study, and for the first time, we showedthe secretion of ANLN in oral cancer. ANLN was essential to growth, proliferation, and invasion oforal cancercells. These traits are the same as the typical hallmarks of cancer. Unlimited expansion and growth without contactinhibition is a great advantage of cancer cells [18]. Cancer cells can continue to grow if only there is sufficientenergy. Metastasis to distant sites is a cause of death of cancer patients. In cancer, cells can flow out of the primary siteby invading the surrounding vasculature or lymphatic vessels [19–21]. Tumor cells secrete matrix metalloproteases to degrade the extracellular matrix and alter the permeability of blood vessels, which leads to leakage of blood vessels. This is an important mechanism, and many drugs that ameliorateblood vessel leakage arebeing developed [22, 23]. In this study, knockdown of ANLN suppressed oral cancercell invasion, which suggests that ANLN might be a new target for drug development. Resistance to apoptosis is evolutionarily conserved amongtumors. However, this mechanism is employed by chemoradiation therapy [24, 25]. In the clinic, chemotherapy drugs can directly cause cell apoptosis or increase the sensitivity of tumor cells to radiation. ANLN displayed antagonistic activity to apoptosis in this study. This suggests that knockdown or knockout of ANLN might be a good way to induce cell apoptosis in oral cancer.

The complete life cycle of cells includes the G1, S, G2, and M phases. In cancer, this cycle is shortened, and cell division is accelerated. This leads to arapidincrease inthenumber of cells in a short time [26, 27]. In this study, knockdown of ANLN did not significantly block the transition from theG1 phase to the S phase in oral cancer. It is possible that ANLN might not be a checkpoint that controlsthe cell cycle in oral cancer.

Overall, the above data show that ANLN plays promotes oral cancer progression.However, what is the molecular mechanismby whichANLN promotes oral cancer? The present data demonstrated that signaling molecules, including PI3K, PDK1, Akt and mTOR, were regulated by ANLN in oral cancer. These molecules are critical members of the PI3K/mTOR signaling pathway. PI3K/mTOR signaling is an important signaling pathway in development and homeostasis [28]. Abnormal activation of PI3K/mTOR signaling often causes serious diseases, especially cancer [28, 29]. In cancer, activation of PI3K/mTOR signaling results in cell proliferation, prolongs cell survival, shortens the cell cycle and suppresses cell apoptosis. For example, PI3K/mTOR signaling is activated in lung cancer, and drugs targeting this signaling pathway are being investigated [30]. In liver cancer, cell apoptosis was inhibited through the activation of PI3K/mTOR signaling [31]. In oral cancer, PI3K/mTOR signaling also contributes to progression and deterioration. Inhibitors ofPI3K/mTOR signaling could increase the radiosensitivity of oral cancer cells [32, 33]. Furthermore, ANLN affected PI3K/mTOR signaling in previous studies. Mutations in ANLN resulted in dysregulated PI3K/mTOR signaling in podocytes [1]. ANLN regulated PI3K/Akt signaling in lung cancer and promoted cancer progression [34]. In this study, our data indicate that ANLN could also affect the activation of PI3K/mTOR signaling in oral cancer. These findings increase our knowledge about the role of ANLN in oral cancer and the underlying mechanism. However, more experiments are necessary to linkANLN and PI3K/mTOR signaling. The identification of the clinical importanceof ANLN in a large cohort of oral cancer specimens will further support the data from TCGA database. In addition, the role of ANLN in a xenograft mouse model in vivo will be examined in the future.

## Conclusions

In summary, ANLN was overexpressed and associated with patient prognosis in oral cancer. ANLN regulated cell growth, proliferation, invasion, and apoptosis *in vitro*. ANLN participated in activation of PI3K/mTOR signaling. Patients with oral cancer will benefit from this study in the future.

## Data Availability

The datasets used and/or analyzed during the current study are available from the corresponding author on reasonable request.

## Abbreviations

ANLN: Anillin actin binding protein
mTOR: Mammalian target of rapamycin
TCGA: The cancer genome atlas
DMEM: Dulbecco’s modification of Eagle’s medium
QPCR: Realtime quantitative polymerase chain reaction assay
ECL: Efficient chemiluminescence kit
CCK-8: Cell counting kit-8
RNAi: RNA interference
